# A Text-Book of Legal Medicine and Toxicology

**Published:** 1903-10

**Authors:** 


### Books and Magazines

A Text-Book of Legal Medicine and Toxicology.—Edited by Frederick Peterson, M. D., Chief of Clinic, Nervous Department of the College of Physicians and Surgeons, New York; and Walter S. Haines, M. D., Profesor of Chemistry, Pharmacy, and Toxicology, Rush Medical College, in affiliation with the University of Chicago. Two imperial octavo volumes of about 750 pages each, fully illustrated. Philadelphia, New York, London; W. B. Saunders & Co. 1903. Per volume: Cloth, $5.00, net; Sheep or Half Morocco, $6.00 net.

This work presents to the medical and legal professions a comprehensive survey of forensic medicine and toxicology in moderate compass, yet with sufficient detail. For convenience of reference the treatise has been divided into two sections, part I and part II, the latter being devoted to toxicology, and all other portions of legal medicine in which laboratory investigation is an essential feature. The introduction, of expert evidence, is especially instructive to the lawyer and medical expert. Medico-legal post-mortem examinations comprise an interesting chapter at the beginning of the volume. The Bertillon and Greenleaf-Smart systems of identification are concisely and intelligently described, and the advantages of each stated.

The chapters on the signs of death deal with the various causes and effects in a most comprehensive manner. An instructive and important chapter is devoted to the destruction and attempted destruction of the human body by fire and chemicals. A chapter not usually found in works on legal medicine is that on medico-legal relations of the X-ray, which throws out valuable suggestions and encompasses all the facts now known on the subject. In dealing with the insane, the various State laws regarding same, we find a chapter which every practitioner of medicine should read.

In fact the entire work is overflowing with matters of the utmost importance and expresses clearly, concisely, and accurately the very latest opinions on all branches of forensic medicine and toxicology.

